# P-336. Validation of a Badge-Based Automated Hand Hygiene Monitoring System

**DOI:** 10.1093/ofid/ofae631.539

**Published:** 2025-01-29

**Authors:** Palak Patel, Molly Steele, Jennifer Incesti, Vera Chu, R Marrs, Patricia D Zuccaro, Allison H Bartlett, Emily Landon

**Affiliations:** The University of Chicago, Chicago, Illinois; University of Chicago, Chicago, Illinois; University of Chicago, Chicago, Illinois; UChicago Medicine, Chicago, Illinois; University of Chicago Medicine, Chicago, Illinois; The University of Chicago Medicine, Chicago, Illinois; University of Chicago Comer Children's Hospital, Chicago, IL

## Abstract

**Background:**

Hand Hygiene (HH) is the cornerstone behavior in preventing healthcare-associated infections (HAIs) yet accurately monitoring HH remains a challenge. We present the validation process and results for a popular badge-based automated monitoring system in the setting of existing aggregate, automated HH monitoring.

Accuracy of Unique Actions of Biovigil Monitoring System by Unit

**Figure:**
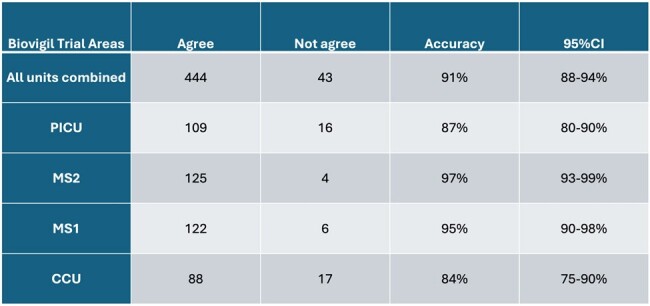

Accuracy of Biovigil monitoring system compared to observation by IPC Team in behavioral validation phase by unit

**Methods:**

University of Chicago Medicine Hyde Park Campus (UCM) is an 800 bed academic medical center and Ingalls Memorial Hospital (IMH) is a 200 bed community hospital. The Biovigil HH monitoring system (Ann Arbor, MI) was trialed on 4 units: IMH 35 bed med/surg unit (MS1), IMH 8 bed cardiac care unit (CCU), UCM 36 bed med surg unit (MS2), and UCM 19 bed Pediatric intensive care unit (PICU). Validation utilized planned path and behavioral validation techniques previously reported by the authors (Landon ICHE 2016). Participation by employees was voluntary.

Accuracy of Unique Actions of Biovigil Monitoring System by Event

**Figure:**
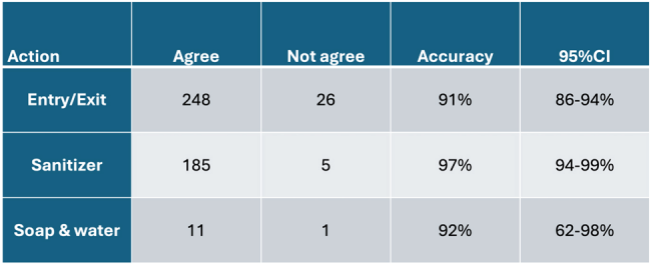

Accuracy of Biovigil monitoring system compared to observation by IPC Team in behavioral validation phase by event

**Results:**

During the planned path, investigators performed 336 unique actions and observations to test functionality. This identified issues with installation of soap use detectors on one unit. Once these were adjusted, overall accuracy was 99.7% (MS1 100%, MS2 100%, CCU 100%, PICU 98.8%). During behavioral validation, investigators observed 259 unique behaviors and compared with system raw data found an overall accuracy of 91% (MS1 95%, MS2 97%, CCU 84%, PICU 87% with 95% CI) as shown in table 1. Accuracy for detecting entry or exit was 91% (95%CI 86-94%), sanitizer use 97% (95%CI 94-99%), and soap and water use 92% (95%CI 62-98%) as seen in table 2. Monthly HH rates measured by the existing monitoring system (only UCM) before and during the trial system validation are shown in table 3.

Monthly HH Rates

**Figure:**
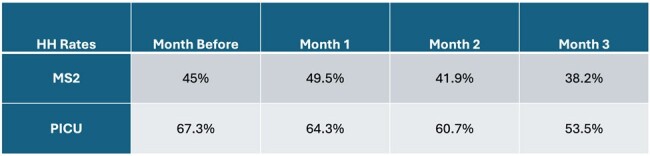

Monthly HH rates measured by the existing monitoring system (only UCM) before and during the trial system validation

**Conclusion:**

The trial HH monitoring system was more accurate on floor units than intensive care units but performed similarly and adequately across two different types of hospitals. There is insufficient data to evaluate for accuracy in measuring soap and water compliance. While HH compliance as measured by the existing system increased initially after installation of the trial system, this was not sustained and may be related to attrition in badge wearing for the trial system.

**Disclosures:**

**Allison H. Bartlett, MD, MS**, CVS/Caremark: Advisor/Consultant

